# Abordagem Híbrida de Extração e Implantação Simultâneas de Marca-passo sem Eletrodo em um Caso de Endocardite por Eletrodo Transvenoso

**DOI:** 10.36660/abc.20220091

**Published:** 2023-02-13

**Authors:** Helder Santos, André Grazina, Mariana Santos, Paulo Osório, Guilherme Portugal, Ana Lousinha, Bruno Valente, Pedro Silva Cunha, Mário Oliveira

**Affiliations:** 1 Departamento de Arritmologia Serviço de Cardiologia Centro Hospitalar Universitário Lisboa Central Lisboa Portugal Departamento de Arritmologia - Serviço de Cardiologia , Centro Hospitalar Universitário Lisboa Central , Lisboa – Portugal; 2 Serviço de Cardiologia Centro Hospitalar Barreiro Montijo E.P.E Barreiro Portugal Serviço de Cardiologia – Centro Hospitalar Barreiro Montijo E.P.E ., Barreiro – Portugal

**Keywords:** Equipamentos e Provisões/instrumentação, Marca-Passo Artificial/efeitos adversos, Marca-Passo Sem Fio, Estimulação Cardíaca Artificial/métodos, Bradicardia/complicações, Reimplante, Método de PISA

## Introdução

O tratamento da bradicardia sintomática baseia-se no método de estimulação endocárdica transvenosa. ^1^ Entretanto, os dispositivos cardíacos eletrônicos implantáveis (DCEI) estão associados a um risco potencial de complicações, incluindo infeções, com uma taxa estimada de 0,5% com implantes primários e 1–7 % com intervenções secundárias. ^1^ Infecções por DCEI estão associadas a maior tempo de internação hospitalar, maiores custos clínicos e taxas de mortalidade. ^2 , 3^ De acordo com as diretrizes, a endocardite infecciosa relacionada ao DCEI implica a remoção completa do sistema, seguida de um período sem terapia intravascular. ^1 - 4^ Entretanto, a maioria dos pacientes necessita de reimplante de DCEI, o que sabidamente está associado a um risco de reinfecção entre 2 e 11%, principalmente nos casos com remoção apenas parcial do dispositivo inicial. ^5^

A técnica PISA é um procedimento percutâneo utilizado com sucesso na extração de eletrodos do DCEI. ^6^ Essa técnica inicia-se com a identificação da porção proximal do eletrodo. Em seguida, é realizado um desbridamento ao longo do eletrodo para atingir o local da inserção venosa. A seguir, uma bainha dilatadora de polipropileno é inserida e avançada externamente até o eletrodo em movimentos rotacionais, mantendo-se uma leve tração. Tais movimentos irão resultar na liberação de aderências ao redor do eletrodo, permitindo, após o avanço total da bainha, a remoção total do eletrodo. ^7^

### Caso clínico

Paciente do sexo feminino, 86 anos, ex-tabagista, com história de hipertensão, hiperlipidemia, diabetes, obesidade e hiperuricemia, deu entrada no pronto-socorro com quadro de dor torácica atípica e febre dez meses após implante de marca-passo DDDR por bloqueio atrioventricular de terceiro grau. A primeira avaliação clínica detectou sinais de infecção local na mão esquerda, onde a paciente havia sido submetida a intervenção cirúrgica dois meses antes. Os exames de sangue sugeriram uma infecção sistêmica, apesar das hemoculturas negativas. O ecocardiograma transtorácico mostrou fração de ejeção do ventrículo esquerdo normal, sem doença valvar significante e sem identificação de vegetações ou complicações locais sugestivas de endocardite. Ainda assim, considerando a suspeita de endocardite, foi realizado um ecocardiograma transesofágico duas semanas após a internação inicial, revelando duas massas no átrio direito, aderidas ao eletrodo ventricular, com dimensões máximas de 21x7 mm. Antibioticoterapia com vancomicina e ceftriaxona foi iniciada e mantida por 35 dias, enquanto as hemoculturas permaneceram estéreis. Ela foi então encaminhada ao nosso centro para extração de eletrodo pela técnica PISA.

**Figura 1:**
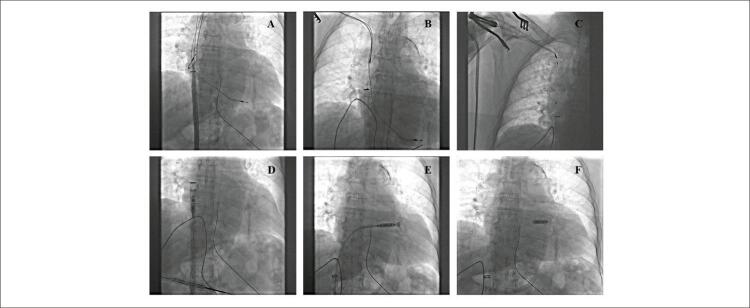
– Imagens do procedimento, mostrando a retirada do marca-passo transvenoso e a implantação do marca-passo sem eletrodos, posicionado no septo médio-ventricular (F).

Além disso, a paciente era totalmente dependente do ritmo do marca-passo. Portanto, para promover a sincronização atrioventricular, decidimos implantar o marca-passo sem eletrodo Micra AV (Medtronic Inc., Minneapolis, MN, EUA), habilitando um modo de estimulação VDD. A extração do marca-passo e o implante do Micra AV foram realizados em procedimento simultâneo. Implantou-se inicialmente marca-passo intracardíaco sem eletrodo, conforme recomendação de treinamento do fabricante, via acesso femoral esquerdo. Um cateter de entrega Micra ^TM^ Delivery (105 cm de comprimento) inserido na bainha introdutora Micra ^TM^ (diâmetro externo de 27 Fr) foi desviado para o átrio direito sem dificuldade. Um operador foi responsável por manter o cateter de entrega no átrio direito, enquanto outros dois operadores iniciaram a extração do marca-passo. A remoção completa de ambos os eletrodos foi obtida pelo método PISA ( Figura 1 ). Nessa fase, a implantação adequada do marca-passo intracardíaco sem fio permitiu alcançar uma posição estável no septo médio-ventricular. Todo o procedimento ocorreu sem intercorrências.

No dia seguinte, os parâmetros estáveis do Micra AV foram confirmados e otimizados, o acesso femoral foi verificado e não apresentou complicações. A paciente recebeu antibioticoterapia por mais 12 dias. Um ecocardiograma transesofágico foi realizado uma semana após o procedimento, sem sinais de endocardite. No seguimento de um mês, os parâmetros do marca-passo estavam estáveis, com detecção atrial precisa e estimulação ventricular de 100%, e a paciente permanecia assintomática, sem intercorrências.

## Discussão

Os dispositivos intracardíacos sem eletrodos são agora uma alternativa segura e eficaz aos marca-passos transvenosos, principalmente em pacientes com infecções anteriores relacionadas ao dispositivo, problemas de acesso venoso, extração anterior de eletrodos e comorbidades, incluindo insuficiência renal em estágio terminal e diabetes. ^8 , 9^ A ausência de um bolso subcutâneo ou eletrodos transvenosos evitam o risco potencial de infecção associado a esses componentes. ^8 , 10^ O fato do sistema Micra ter uma pequena área de superfície, ser inteiramente endovascular (com encapsulamento) e submetido a maior turbulência sanguínea, velocidade e pressão também pode favorecer um menor risco de infecção. ^9^

Pacientes com infecções sistêmicas têm pior prognóstico em curto e longo prazos e, mesmo após a extração completa do dispositivo e resolução da infecção sistêmica, observam-se altas taxas de mortalidade em um ano, entre 20% e 35%, ^3^ várias vezes associada a um risco ainda maior de reinfecção. É particularmente importante selecionar o procedimento mais adequado, e é por isso que a escolha de um dispositivo intracardíaco sem eletrodo parece garantir uma importante alternativa de abordagem para pacientes dependentes de marca-passo. El-Chami et al., ^8^ demonstraram que o implante do dispositivo Micra é seguro após infecção prévia de marca-passo, pois não foi observada infecção causada pelo Micra e não foram detectadas infecções sistêmicas com necessidade de remoção do dispositivo durante o seguimento. Além disso, a capacidade do dispositivo intracardíaco sem eletrodos de fornecer sincronia atrioventricular é vantajosa, pois pode evitar alguns dos efeitos deletérios associados à estimulação de câmara única. ^11^

O momento ideal para realizar o reimplante é desconhecido e controverso. A literatura anterior ^8 , 12^ descreve apenas alguns casos de procedimento simultâneo, pois a maioria dos operadores prefere concluir a extração e realizar o reimplante alguns dias depois. No entanto, as recomendações sugerem que o reimplante deve ocorrer pelo menos 72 horas após a extração e verificação de hemoculturas negativas. ^13^ Curiosamente, uma recente metanálise concluiu que o reimplante após 72 horas estava associado a um maior risco de reinfecção do novo sistema cardíaco. ^14^ O reimplante em procedimento simultâneo de um novo sistema de estimulação e extração de eletrodo já se mostrou viável, sem aumentar as taxas de complicações. ^13^ Nossa decisão de realizar as duas técnicas simultaneamente foi tomada de acordo com uma avaliação de risco-benefício. Considerando a evolução clínica durante a hospitalização, a idade e a presença de várias comorbilidades, o risco intrínseco de infeção associado aos procedimentos repetidos e todas as potenciais complicações, um procedimento único pareceu ser a melhor alternativa de abordagem. Além disso, as evidências da literatura ^8^ sobre a segurança do dispositivo intracardíaco sem eletrodo em pacientes com infecções preexistentes reforçam essa suposição e, portanto, a abordagem utilizada por nossa equipe.
