# Supplementary material for: Abordagem Híbrida de Extração e Implantação Simultâneas de Marca-passo sem Eletrodo em um Caso de Endocardite por Eletrodo Transvenoso
Source: Arq Bras Cardiol. 2023 Feb 13;120(2):e20220091. [Article in Portuguese] doi: 10.36660/abc.20220091 (PMC10263459; doi:10.36660/abc.20220091)
Supplement: Translation

# Translation: A Hybrid Approach of Simultaneous Extraction and Leadless Pacemaker Implantation in a Transvenous Lead Endocarditis Case

**Authors:** Helder Santos, André Grazina, Mariana Santos, Paulo Osório, Guilherme Portugal, Ana Lousinha, Bruno Valente, Pedro Silva Cunha, Mário Oliveira

**Affiliations:** 1 Portugal Departamento de Arritmologia - Serviço de Cardiologia, Centro Hospitalar Universitário Lisboa Central, Lisboa – Portugal; 2 Portugal Serviço de Cardiologia – Centro Hospitalar Barreiro Montijo E.P.E., Barreiro – Portugal

**Keywords:** Equipment and Supplies/instrumentation, Pacemaker, Artificial/adverse effects, Leadless Pacemaker, Cardiac Pacing, Artificial/methods, Bradycardia/complications, Replantation, Method PISA

## Introduction

Symptomatic bradycardia treatment is based on the transvenous method of endocardial pacing.^1^However, cardiac electronic implantable devices (CEID) are associated with a potential risk of complications, including infections, with an estimated rate of 0.5% with primary implants and 1–7 % with secondary interventions.^1^CIED infections are associated with longer hospital length of stay, higher clinical costs and mortality rates.^2 , 3^According to guidelines, infective endocarditis related to CIED implicates complete removal of the system, followed by a period without intravascular therapy.^1 - 4^Nevertheless, most of the patients require CIED reimplantation, which is known to be associated with a risk of reinfection between 2 and 11%, particularly in cases with only partial removal of the initial device.^5^

The PISA technique is a percutaneous procedure used with success in lead extraction of the CIED.^6^This technique starts with the identification of the proximal portion of the lead. Then, a debridement is performed along the lead to achieve the site of venous insertion. Subsequently, a polypropylene dilator sheath is inserted and advanced externally up to the lead in rotational movements, while maintaining a light traction. Such movements will cause the release of adhesions around the lead, allowing, after the full advancement of the sheath, the entire lead removal.^7^

### Clinical case

An 86-year-old female patient, a former smoker with a history of hypertension, hyperlipidemia, diabetes, obesity and hyperuricemia was admitted to the emergency room with atypical chest pain and fever ten months after a DDDR pacemaker implantation due to a third-degree atrioventricular block. The first clinical evaluation detected signs of local infection in the left hand, where the patient had a surgical intervention two months before. Blood tests suggested a systemic infection, despite negative blood cultures. A transthoracic echocardiogram showed normal left ventricular ejection fraction, without any significant valvular disease, and without the identification of vegetations or local complications suggesting endocarditis. Yet, considering the suspicion of endocarditis, a transesophageal echocardiogram was performed two weeks after the initial hospital admission, revealing two masses in the right atrium, attached to the ventricular lead, with maximum dimensions of 21x7 mm. Antibiotic therapy with vancomycin and ceftriaxone was initiated and continued for 35 days, while blood cultures remained sterile. She was then referred to our center for lead extraction using the PISA technique.

Moreover, the patient was totally dependent on the pacemaker rhythm. Therefore, to promote atrioventricular synchronization, we decided to implant the Micra AV (Medtronic Inc., Minneapolis, MN, USA) leadless pacemaker, enabling a VDD pacing mode. Pacemaker extraction and the Micra AV implantation were performed in a simultaneous procedure. An intracardiac leadless pacemaker was implanted initially according to the manufacturer’s training recommendation via the left femoral access. A Micra^TM^Delivery Catheter (105-cm-long) inserted in the Micra^TM^introducer sheath (27-Fr outer diameter) was deflected into the right atrium without difficulty. One operator was responsible to maintain the delivery catheter in the right atrium, while two other operators started the pacemaker extraction. Complete removal of both leads was obtained with the PISA method ( Figure 1 ). At this stage, a suitable deployment of the intracardiac leadless pacemaker allowed a stable position to be reached in the mid-ventricular septum. The entire procedure occurred without complications.

**Figure 1:** – Images of the procedure, showing the extraction of the transvenous pacemaker and the implantation of the leadless pacemaker, positioned in the mid-ventricular septum (F).

On the next day, Micra AV stable parameters were confirmed and optimized, the femoral access was checked and did not show any complications. The patient received antibiotic therapy for 12 more days. A transesophageal echocardiogram was performed one week after the procedure, without any signs of endocarditis. At the one-month follow-up, the pacemaker parameters were stable, with 100% accurate atrial sensing and ventricular pacing, and the patient remained asymptomatic, without complications.

## Discussion

Intracardiac leadless devices are now a safe and effective alternative to transvenous pacemakers, namely in patients with previous device-related infections, venous access issues, previous lead extraction, and comorbidities, including end-stage renal failure and diabetes.^8 , 9^The absence of a subcutaneous pocket or transvenous leads prevent the potential risk of infection associated with these components.^8 , 10^The fact that the Micra system has a small surface area, is entirely endovascular (with encapsulation) and submitted to higher blood turbulence, velocity and pressure may also favor a lower infection risk.^9^

Patients with systemic infections have worse short- and long-term prognosis, and, even when complete device extraction has been accomplished and the systemic infection was resolved, high mortality rates are observed at one year, between 20% and 35%,^3^several times associated with an even higher risk of reinfection. It is particularly important to select the most suitable procedure, and that is why choosing an intracardiac leadless device seems to guarantee an important alternative approach for dependent-pacemaker patients. El-Chami, et al.^8^showed that Micra implantation is safe after a previous pacemaker infection, since no Micra infection was observed and systemic infections requiring device removal were not detected during the follow-up. Also, the ability of the intracardiac leadless device to provide atrioventricular synchrony is advantageous, since it may prevent some of the deleterious effects associated with a single-chamber pacing.^11^

The optimal timing to perform the reimplantation is unknown and controversial. Previous literature^8 , 12^described just a few cases of a simultaneous procedure, since most of the operators prefer to complete the extraction and perform the reimplantation a few days later. Nonetheless, the recommendations suggest that reimplantation should occur at least 72 hours after the extraction and documentation of negative blood cultures.^13^Curiously, a recent meta-analysis concluded that the reimplantation after 72 hours was associated with a higher risk of reinfection of the new cardiac system.^14^Reimplantation in a simultaneous procedure of a new pacing system and lead extraction has already proved to be feasible, without increasing complication rates.^13^Our decision to make both techniques simultaneously was made according to a risk-benefit balance evaluation. Considering the clinical evolution during the hospitalization, age and the presence of several comorbidities, the intrinsic risk of infection associated with repeated procedures and all the potential complications, a single procedure seemed to be the best alternative approach. Moreover, literature evidence^8^of the safety of intracardiac leadless device in patients with pre-existing infections reinforces this assumption and, therefore, the approach used by our team.
